# Prosocial Behaviors during School Activities among Child Survivors after the 2011 Earthquake and Tsunami in Japan: A Retrospective Observational Study

**DOI:** 10.1371/journal.pone.0113709

**Published:** 2014-11-21

**Authors:** Masahide Usami, Yoshitaka Iwadare, Kyota Watanabe, Masaki Kodaira, Hirokage Ushijima, Tetsuya Tanaka, Maiko Harada, Hiromi Tanaka, Yoshinori Sasaki, Seiko Okamoto, Keisuke Sekine, Kazuhiko Saito

**Affiliations:** 1 Department of Child and Adolescent Psychiatry, National Center for Global Health and Medicine, Kohnodai Hospital, Chiba, Japan; 2 Department of Child Mental Health, Imperial Gift Foundation, Aiiku Hospital, Tokyo, Japan; 3 Division of Neuropsychiatry, Department of Neuroscience, Yamaguchi University Graduate School of Medicine, Yamaguchi, Japan; 4 Institute of Women's Health, Tokyo Women's Medical University, Tokyo, Japan; 5 Department of Psychiatry, Tokyo Metropolitan Tama Medical Center, Tokyo, Japan; University of California, San Francisco, United States of America

## Abstract

**Background:**

The 2011 Japan massive tsunami traumatized many children. The aim of this study was to assess changes in strengths and difficulties experienced in home and school by among surviving children after the 2011 tsunami, in comparison with published normal Japanese data.

**Methods:**

In November 2012 (20 months after the disaster) and September 2013 (30 months after the disaster), the Strengths and Difficulties Questionnaire (SDQ), a questionnaire on children's strengths and difficulties in home and school activities, were distributed to 12,193 and 11,819 children, respectively. An effective response of children 20 months and 30 month after the disaster was obtained in 10,597 children (86.9%), and 10,812 children (91.4%), respectively. The SDQ scores evaluated by parents and teachers were compared with published normal Japanese SDQ scores.

**Results:**

The SDQ scores (emotional problems, conduct problems, hyperactivity/inattention, peer relationship problems, and total difficulty score) evaluated by parents of children in the 4th to 9th grade who were evaluated after 30 and 20 months were significantly high compared with the published normal data of children without traumatic experiences (all *P*<0.001). The SDQ scores (prosocial behavior) evaluated by teachers of children in the 4th to 9th grade who were evaluated after 30 and 20 months were significantly low compared with the published normal data of children without traumatic experiences (all *P*<0.001).

**Conclusions:**

This study showed that the experience of the disaster affected those children with prosocial behaviors towards teachers and friends at school. However, no significant changes (in their prosocial attitude) had been seen at home, where they continued to keep their respect and caring feelings for parents. These results indicate that for accurate diagnosis, clinicians should not only evaluate these children's daily activities at home but also try to objectively assess their daily activities at school.

## Introduction

On March 11, 2011, Japan was struck by a huge earthquake and tsunami. The tsunami caused tremendous damage and victimized many children [1]–[8]. In the event of any disaster, post-traumatic stress disorder (PTSD) should be considered most carefully by health care providers [1]–[23]. Traumatic symptoms tend to spontaneously disappear over time; thus, the morbidity of PTSD is dependent on time, the subjects, and the diagnostic methods used.

The diagnostic criteria for PTSD in the Diagnostic and Statistical Manual of Mental Disorders, 5^th^ edition (DSM-5), specify that patients experience significant difficulties in their daily lives because of their traumatic symptoms [24]. The PTSD criteria of DSM-5 include childhood traumatic symptoms, such as repetitive play and nightmares. To plan a treatment strategy, it is very important to evaluate these children and establish a diagnosis. Furthermore, for a diagnosis of psychiatric disorders, clinicians should evaluate the daily activities of such children [25]–[27].

We collected information on the daily activities of children who survived the 2011 Japanese earthquake and tsunami [1]–[6]. In a preliminary study conducted 8 months after the 2011 disaster, we identified relationships between post-traumatic symptoms and sex, age, house damage, sleep duration, evacuation experience, and bereavement experience, using the Posttraumatic Stress Symptoms for Children 15 items (PTSSC-15) tool [3], [4]. PTSSC-15 is a self-completion questionnaire on stress reactions of children after disasters. In the second study conducted 20 months after the earthquake and tsunami, we used the Strengths and Difficulties Questionnaire (SDQ) to collect information on the strengths and difficulties faced by parents and teachers while dealing with the traumatic symptoms of children who survived the disaster [2], [28]. SDQ has been widely used in the pediatric clinical field in Japan [29], [30]. The standard distribution of SDQ scores among Japanese children was published by the Ministry of Health, Labour and Welfare [31]. Japanese clinicians use these data to compare the SDQ scores evaluated by patients with the published normal SDQ score [30]. The second study showed that the difficulties faced by parents and teachers while dealing with child survivors of the disaster were not significantly relative with these children's traumatic symptoms [2]. Thus, clinicians should not only evaluate traumatic symptoms but also try to objectively evaluate whether there are difficulties in daily activities due to the post-traumatic symptoms [2].

In the present study, which was conducted 30 months after the earthquake and tsunami, we used PTSSC-15 and SDQ to collect information similar to that collected in the study conducted 20 months after the disaster. Previous studies did not elucidate the strengths and difficulties of child survivors compared with those of children without traumatic experiences.

The aim of this study was to evaluate the strengths and difficulties in child survivors' daily activities experienced by their parents and teachers when dealing with the children 20 and 30 months after the major disaster. Our main hypothesis was that the traumatic experience undermined the children's strengths, emotional problems, conduct problems, hyperactivity/inattention, and peer relationships.

## Materials and Methods

### Study Design and Setting

This study involved the observation of statistical associations between traumatic symptoms among children and the aftermath of the 2011 Japanese earthquake and tsunami. Ishinomaki City is the second largest city (population 162,822) in Miyagi Prefecture, Japan. As of February 15, 2012, the death toll of the disaster in this city was 3182, and 557 people were missing. The total number of collapsed houses and buildings, including half-collapsed houses, was 33,378, and 7298 temporary houses were constructed.

### Recruitment and Participants

The Ethics Committee of the National Center for Global Health and Medicine approved this study. The traumatic groups were elementary school and junior high school students who survived the huge tsunami. The published normal SDQ scores of elementary and junior high school students were considered as the control [30].

The survey of the traumatic group was conducted as part of the school education program by the Board of Education of Ishinomaki City. Survey sheets were distributed among all children who attended five kindergartens, 43 elementary schools, and 21 junior high schools in Ishinomaki City. The survey was carried out in November 2011 (8 months after the 2011 disaster), November 2012 (20 months after the 2011 disaster), and September 2013 (30 months after the 2011 disaster).

The surveys in 2011, 2012, and 2013 were administered in the same manner. First, the Education Committee of Ishinomaki City explained the survey method to the principals of all schools. Subsequently, the teachers distributed a letter explaining the survey, which had been constructed by the Education Committee, to all children and their parents. The letter clearly stated that by filling out the questionnaire, both the student and parents were consenting to participate in the survey. The letter also specified that the survey results would be used to provide psychological care to the children and to facilitate their education at school, and that the results would be published as a medical research article. Written informed consents were not obtained from parents/guardians of children from the elementary school and junior high school. However, informed consent was thus obtained when the students filled out the questionnaire. The Ethics Committee of the National Center for Global Health and Medicine approved this consent procedure.

In November 2011 (8 months after the disaster), PTSSC-15 was distributed to 12,524 children who were enrolled in the municipal schools of Ishinomaki City. A questionnaire on the environmental damage experienced by the children was distributed to their teachers.

In November 2012 (20 months after the disaster) and September 2013 (30 months after the disaster), copies of PTSSC-15 were distributed to 12,193 and 11,819 children enrolled in municipal schools, respectively, and to their teachers.

The parents of the children enrolled in kindergarten and in the 1st to 3rd grade of elementary school were asked to fill the questionnaire with responses of their children. Informed consent for participation in the survey was obtained when the completed questionnaires were received from the children.

Responses were obtained from 12,346 (98.6%) of the 12,524 children (8-month period), 11,124 (91.2%) of the 12,193 children (20-month period), and 11,197 (94.7%) of the 11,819 children (30-month period) to whom PTSSC-15 was sent. An effective response was obtained in 11,639 (92.9% of the 8-month group), 10,597 (86.9% of the 20-month group), and 10,812 (91.4% of the 30-month group) children. Because of anonymity, the effective responses recorded at 30 months had no connection with the effective responses recorded at 20 months. Similarly, the effective responses recorded at 20 months had no connection with the effective responses recorded at 8 months.

Furthermore, a total of 12,193 copies (20 months after the disaster) and 11,819 copies (30 months after the disaster) of the Strength and Difficulties Questionnaire (SDQ) for teachers were distributed among the teachers of the same elementary school (1^st^ to 6^th^ grade) and junior high school students (7^th^ to 9^th^ grade) in Ishinomaki City. SDQ for parents was distributed to 8404 parents of elementary school (4^th^ to 6^th^ grade) and junior high school (7^th^ to 9^th^ grade) students in Ishinomaki City. Twenty months after the disaster, completed SDQs for teachers were obtained from 10,787 (88.4%) teachers. A valid response was obtained from 10,577 (86.7%) teachers. In addition, completed SDQs for parents were obtained from 7308 (87.0%) parents. A valid response was obtained from 7052 (83.9%) parents. Thirty months after the disaster, completed SDQs for teachers were obtained from 11,111 (94.0%) teachers. A valid response was obtained from 10,910 (92.3%) teachers. Finally, completed SDQs for parents were obtained from 8325 (95.6%) parents. A valid response was obtained from 7310 (83.9%) parents.

Conversely, responses to the questionnaire about environmental damage 8 months after the disaster, in connection with all 12,524 children, were obtained from teachers. Table 1 shows data pertaining to sex, age, and damage due to environmental conditions (house damage, evacuation conditions, and bereavement experience) for 11,639 children 8 months after the disaster [3]. When teachers had no information regarding house damage, evacuation conditions, and bereavement experience, the response was recorded as “unknown.”

**Table 1:** Damage to the living conditions of children affected by the 2011 Japan earthquake and tsunami.

Items			N = 11,639	
House damage	No		6986	(60.0%)
	Yes	Total collapse	2243	(19.3%)
		Half-collapse	2354	(20.2%)
		Total	4597	(39.5%)
	Unknown		56	(0.5%)
Evacuation experience	No		8228	(70.7%)
	Yes	Currently living in an evacuation center	90	(0.8%)
		Used to live in an evacuation center	2845	(24.4%)
		Living in temporary housing	976	(8.4%)
		Used to live in temporary housing	51	(0.4%)
		Evacuation experience at least once	3248	(27.9%)
	Unknown		163	(1.4%)
Bereavement experience	No		9241	(79.4%)
	Yes	Father	71	(0.6%)
		Mother	66	(0.6%)
		Brothers and sisters	44	(0.4%)
		Grandfather and grandmother	355	(3.1%)
		Classmates	1498	(12.9%)
		Teacher in-charge	32	(0.3%)
		Others	270	(2.3%)
		At least one bereavement experience	2103	(18.1%)
	Unknown		295	(2.5%)

N, number of cases.

All parents of schoolchildren (aged 7–15 years) for the normative data were recruited countrywide with assistance from the Japanese Ministry of Education, Culture, Sports, Science, and Technology and local government boards of education. That study did not include private schools, national schools, or schools for handicapped children. Morikawa et. al., collected SDQ data before the 2011 Japanese earthquake and tsunami [30]. The parent SDQ to be completed at home was distributed to all parents of schoolchildren (aged 7–15 years) attending mainstream classes in 148 primary schools and 71 secondary schools in the 10 geographical areas making up Japan. The result of that study was published the normative SDQ data for Japanese children [30].

### Measures

A paper-based survey involved asking questions regarding post-traumatic symptoms using a self-report form. The self-report form consisted of PTSSC-15. The difficulties faced by parents and teachers were assessed using the SDQ score.

#### PTSSC-15

PTSSC-15 is a self-rating questionnaire on the stress reactions of children after a disaster. The Post-Traumatic Stress Symptoms 10 (PTSS10) tool has fewer questions and was used as a screening test after the Great Hanshin Earthquake and Niigata–Chuetsu Earthquake; this instrument is widespread in Japan [32], [33]. Five questions that assess important psychosomatic characteristics after a traumatic event (flashbacks, appetite loss, somatic reactions such as headache and abdominal pain, attention deficit, and anxiety) were added to PTSS10; PTSSC-15, which consists of 15 questions, was thus developed in Japan [1]–[3], [6], [34].

Each question is scored at six levels: 0 =  completely disagree, 1 =  mostly disagree, 2 =  partially disagree, 3 =  partially agree, 4 =  mostly agree, and 5 =  completely agree. Higher scores indicate more severe post-traumatic and depressive symptoms. Tominaga et al. demonstrated the reliability and validity of PTSSC-15 in Japanese children and adolescents [35].

#### SDQ

SDQ is a brief behavioral questionnaire for adults aged about 3–16 years [28], [31]. It has several versions: for researchers, clinicians, and educators. Each question is scored at three levels: 0 =  not true, 1 =  somewhat true, and 2 =  certainly true.

SDQ tests 25 attributes, some of which are positive and others negative. These 25 items are divided among five scales: emotional problems (five items), conduct problems (five items), hyperactivity/inattention (five items), peer relationship problems (five items), and prosocial behavior (five items). The scores on the four problematic scales—emotional problems, conduct problems, hyperactivity/inattention, and peer relationship problems—are added up to produce a total difficulty score (based on 20 items).

Higher scores on emotional problems, conduct problems, hyperactivity/inattention, and peer relationship problems as well as a higher total difficulty score indicate a more serious burden for parents or teachers. Conversely, a higher score on prosocial behavior indicates better sociability. Matsuishi et al. demonstrated the reliability and validity of SDQ scores in Japanese children and adolescents [29]. Morikawa et al. reported normative data and the psychometric properties of SDQ among Japanese school-aged children [30].

### Statistical Analysis

Previous study showed that the differences were between SDQ score evaluated by teachers and parents of children in 4^th^ -9^th^ school children [2]. The aim of this study was to discuss these differences between the SDQ scores evaluated by teachers and parents. Therefore, we used the data of children in 4^th^ -6^th^ graders and excluded the data for children in kindergarteners and 1^st^–3^rd^ graders. The Published SDQ normal data were grouped into two categories: 10–12 years of age [4^th^ to 6^th^ graders (elementary school students)] and 13–15 years of age [7^th^ to 9^th^ graders (junior high school students)]. For rating SDQ in specific periods after the disaster, the parents' and teachers' SDQ median scores were determined at those two school levels. The average SDQ score (a child's emotional problems, conduct problems, hyperactivity/inattention, and peer relationship problems; the total difficulty score; and prosocial behavior) in each rater group were calculated separately for the two time points, i.e., 20 and 30 months after the tsunami. The differences between the average SDQ scores recorded at these two time points and the published normal SDQ scores were assessed using a two-factor analysis of variance for each rater and period (time point). Furthermore, these differences were compared using Bonferroni post-hoc tests to compare periods. A significance level of 0.05 was used in the two-sided tests. All calculations were performed using PASW 18.0 J for Macintosh (SPSS, Tokyo, Japan) and Graph Pad Prism 5 for Mac OS× (GraphPad Software, California, USA).

## Results

### Descriptive Information

Participants who were evaluated 20 months after the disaster included 10,597 children (5302 males and 5295 females) who experienced the 2011 Japanese earthquake and tsunami, and those who were assessed 30 months after the earthquake/tsunami included 10,812 children (5434 males and 5,378 females) who experienced the 2011 Japanese earthquake and tsunami. Table 2 shows the sex, PTSSC-15 total score, depression subscale, PTSD subscale, and age of the 11,639 children (5939 males and 5700 females) who were included 8 months after the disaster.

**Table 2:** Characteristics of children affected by the 2011 Japan earthquake and tsunami.

Items		After 8 months			After 20 months			After 30 months	
		N = 11,639			N = 10,597			N = 10,812	
Gender	Male	5939	(51.0%)	5302		(50.0%)	5434		(50.3%)
	Female	5700	(49.0%)	5295		(50.0%)	5378		(49.7%)
Age at the time of the disaster (y) (Mean)		10.9	(SD = 2.7)	10.9		(SD = 2.7)	10.9		(SD = 2.7)
PTSSC-15 Total score (Mean)		20.5	(SD = 14.5)	18.8		(SD = 14.0)	19.7		(SD = 14.2)
PTSSC-15 Depression score (Mean)		**5.4**	(SD = 4.6)	5.0		(SD = 4.4)	4.1		(SD = 3.6)
PTSD score (Mean)		12.6	(SD = 9.1)	11.7		(SD = 8.7)	11.7		(SD = 8.8)

SD, standard deviation; N, number of cases.

### SDQ Scores evaluated by parents after 20 and 30 Months compared with the Published Normal Data

**Figure 1:**
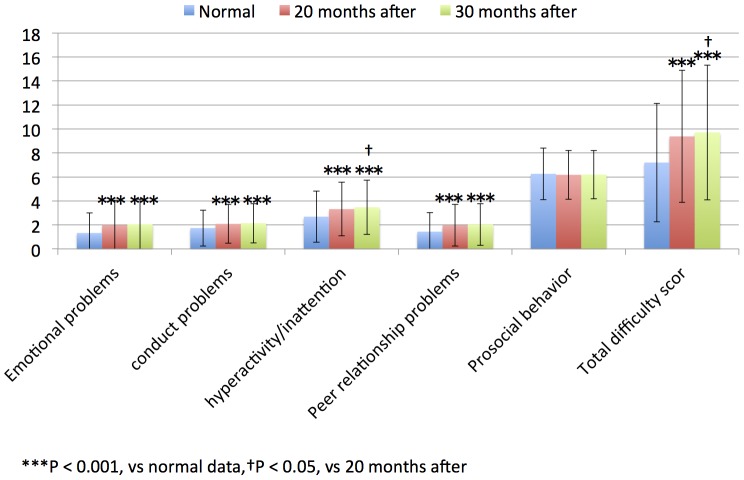
4th–6th grade children SDQ scores of parents in normal data, 20 and 30 months after.

**Figure 2:**
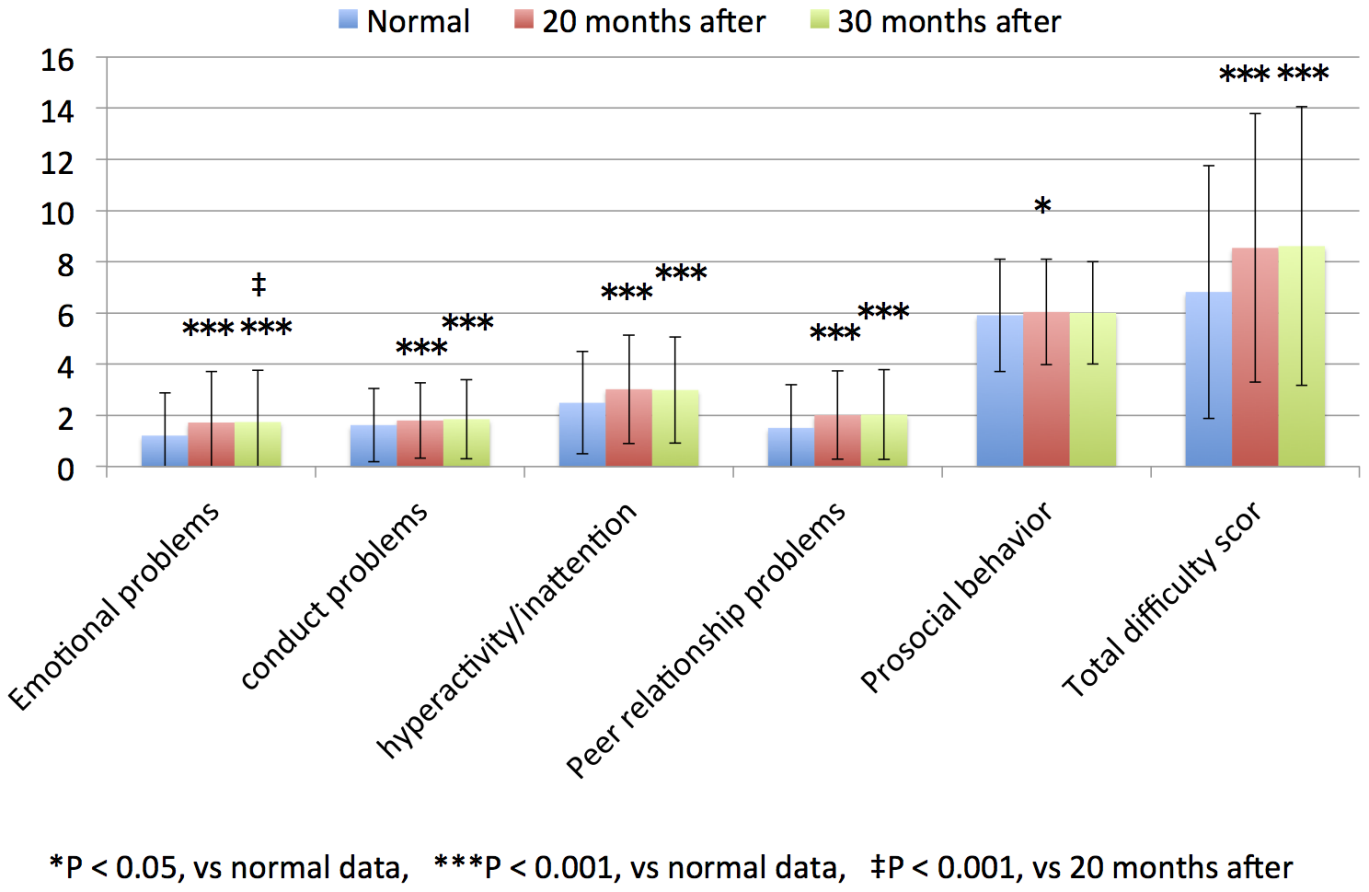
7th–9th grade children SDQ scores of parents in normal data, 20 and 30 months after.

The average SDQ scores evaluated by parents were compared in each grade and period group (Table 3). The SDQ scores (emotional problems, conduct problems, hyperactivity/inattention, peer relationship problems, and total difficulty score) evaluated by parents of children in the 4^th^ to 9^th^ grade groups who were evaluated 30 and 20 months after the disaster were significantly hihg compared with the published normal data of children without traumatic experiences (Table 3, Figs. 1 and 2; all, *P*<0.001).

**Table 3:** Average SDQ scores (by grade, rater, or period).

		Rater	Months after disaster											
			Published normal data			2012 After 20 months			2013 After 30 months				F	P value
			M	SD	N	M	SD	N	M	SD	N			
Emotional problems	4th–6th grade	Parents	1.33	1.67	8584	1.99	2.05	3572	2.06	2.08	3491	Gender × Period	48.19	<0.0001
		Teacher	0.76	1.44	2962	1.01	1.63	3713	0.96	1.60	3547	Period	176.1	<0.0001
												Rater	1460	<0.0001
	7th–9th grade	Parents	1.21	1.68	6267	1.72	1.99	3341	1.74	2.02	3354	Gender × Period	2.897	N.S.
		Teacher	0.64	1.23	1917	1.13	1.84	3632	1.03	1.76	3384	Period	156.0	<0.0001
												Rater	589.0	<0.0001
Conduct problems	4th–6th grade	Parents	1.74	1.5	8584	2.09	1.61	3572	2.14	1.64	3491	Gender × Period	4.112	<0.0001
		Teacher	0.90	1.45	2962	1.15	1.61	3713	1.16	1.56	3547	Period	105.3	<0.0001
												Rater	2008	<0.0001
	7th–9th grade	Parents	1.62	1.43	6267	1.80	1.48	3341	1.85	1.55	3354	Gender × Period	6.759	0.0012
		Teacher	0.81	1.35	1917	1.18	1.63	3632	1.17	1.56	3384	Period	77.88	<0.0001
												Rater	1063	<0.0001
Hyperactivity/inattention	4th–6th grade	Parents	2.69	2.13	8584	3.32	2.23	3572	3.47	2.26	3491	Gender × Period	4.094	0.0167
		Teacher	2.01	2.32	2962	2.71	2.57	3713	2.65	2.54	3547	Period	226.4	<0.0001
												Rater	531.4	<0.0001
	7th–9th grade	Parents	2.49	2.00	6267	3.02	2.12	3341	2.99	2.08	3354	Gender × Period	12.77	<0.0001
		Teacher	1.79	2.04	1917	2.63	2.50	3632	2.65	2.46	3384	Period	208.6	<0.0001
												Rater	228.9	<0.0001
Peer relationship problems	4th–6th grade	Parents	1.44	1.58	8584	1.97	1.73	3572	2.04	1.74	3491	Gender × Period	37.16	<0.0001
		Teacher	1.28	1.75	2962	1.48	1.70	3713	1.43	1.65	3547	Period	125.0	<0.0001
												Rater	362.2	<0.0001
	7th–9th grade	Parents	1.51	1.68	6267	2.01	1.72	3341	2.03	1.75	3354	Gender × Period	13.46	<0.0001
		Teacher	1.34	1.73	1917	1.53	1.72	3632	1.67	1.73	3384	Period	112.4	<0.0001
												Rater	187.4	<0.0001
Prosocial behavior	4th–6th grade	Parents	6.26	2.15	8584	6.18	2.04	3572	6.20	2.02	3491	Gender × Period	45.04	<0.0001
		Teacher	6.48	2.70	2962	5.76	2.59	3713	5.83	2.58	3547	Period	68.26	<0.0001
												Rater	38.50	<0.0001
	7th–9th grade	Parents	5.91	2.20	6267	6.04	2.07	3341	6.00	2.00	3354	Gender × Period	100.8	<0.0001
		Teacher	6.28	2.76	1917	5.45	2.67	3632	5.29	2.71	3384	Period	64.27	<0.0001
												Rater	82.97	<0.0001
Total difficulty scor	4th–6th grade	Parents	7.20	4.94	8584	9.38	5.51	3572	9.71	5.62	3491	Gender × Period	27.24	<0.0001
		Teacher	4.94	5.22	2962	6.35	5.57	3713	6.19	5.50	3547	Period	304.4	<0.0001
												Rater	1747	<0.0001
	7th–9th grade	Parents	6.82	4.94	6267	8.54	5.24	3341	8.61	5.44	3354	Gender × Period	0.529	N.S.
		Teacher	4.58	4.79	1917	6.47	5.77	3632	6.53	5.49	3384	Period	262.0	<0.0001
												Rater	791.4	<0.0001

M, mean; SD, standard deviation; N, number of cases; N.S., not significant.

The SDQ scores (prosocial behavior) evaluated by parents of children in the 4^th^ to 6^th^ grade groups who were evaluated 20 and 30 months after the disaster did not differ with the published normal data of children without traumatic experiences (Table 3, Fig. 1) The SDQ scores (prosocial behavior) evaluated by parents of children in the 7^th^ to 9^th^ grade groups who were evaluated 20 months after the tsunami were significantly high compared with the published normal data of children without traumatic experiences (Table 3, Fig. 2; *P*<0.05).

### SDQ Scores evaluated by teachers 20 and 30 Months after the Disaster Compared with Published Normal Data

The average SDQ scores by teachers were compared in each grade and period group (Table 3).

**Figure 3:**
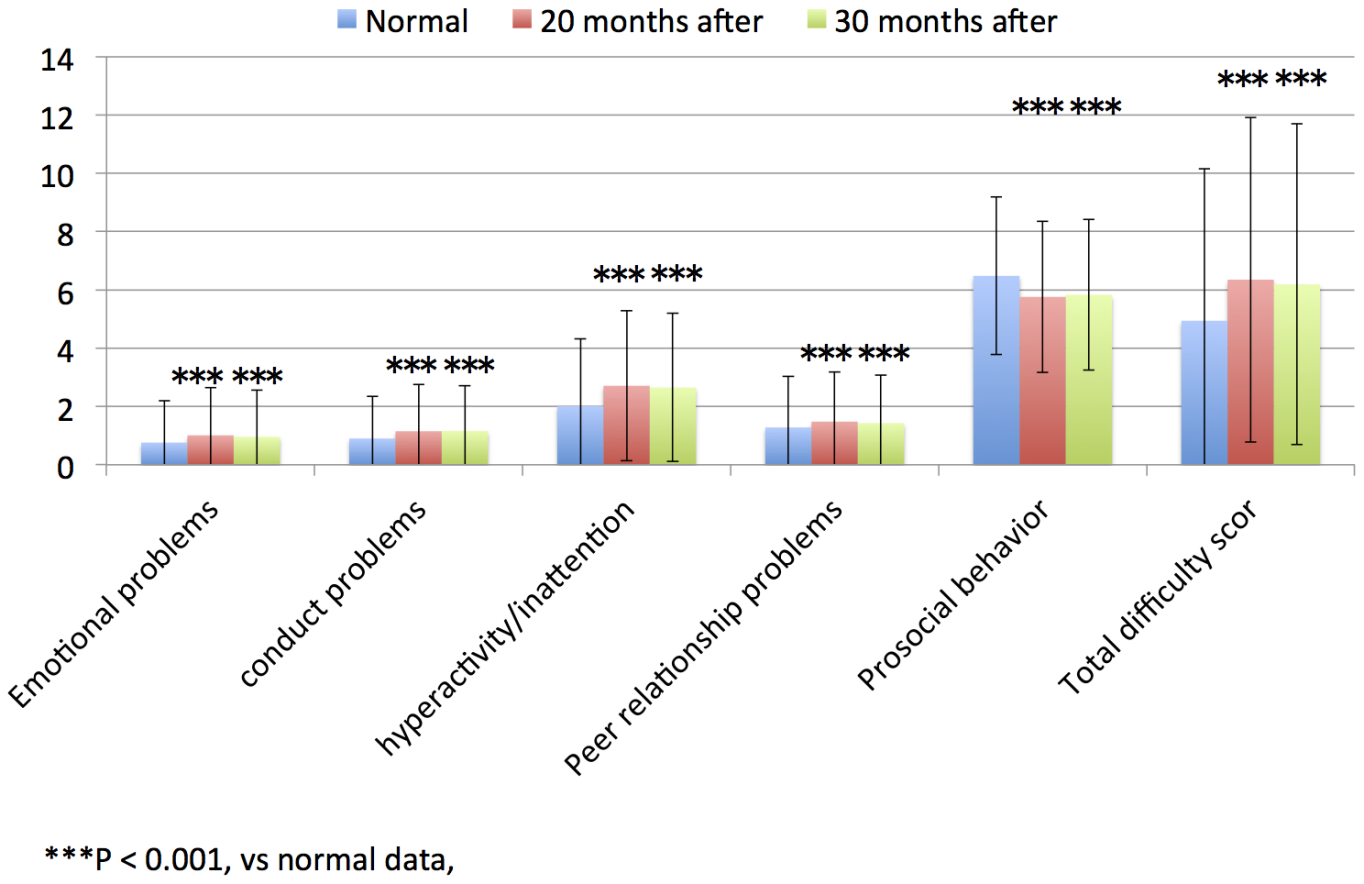
4th–6th grade children SDQ scores of teachers in normal data, 20 and 30 months after.

**Figure 4:**
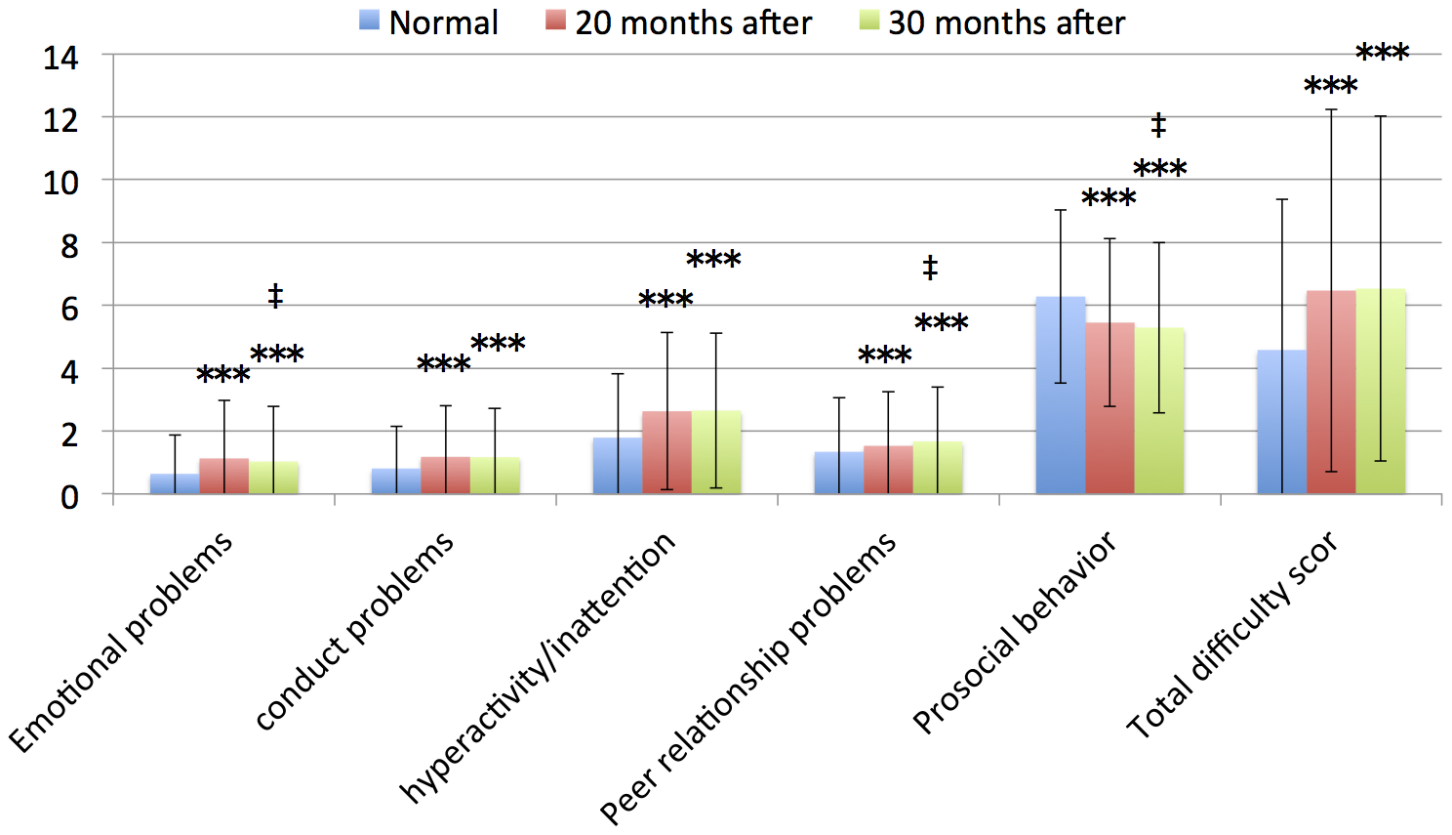
7th–9th grade children SDQ scores of teachers in normal data, 20 and 30 months after.

The SDQ scores (emotional problems, conduct problems, hyperactivity/inattention, peer relationship problems, total difficulty score, and prosocial behavior) evaluated by teachers of children in the 4^th^ to 9^th^ grade groups who were evaluated 30 and 20 months after the tsunami were significantly high with the published normal data of children without traumatic experiences (Table 3, Figs. 3 and 4; all, *P*<0.001). The SDQ scores (prosocial behavior) evaluated by teachers of children in the 7^th^ to 9^th^ grade groups who were evaluated 20 months after the tsunami were significantly low compared with the published normal data of children without traumatic experiences (Table 3, Fig. 3 and 4; *P*<0.001).

## Discussion

The results of this study showed that the SDQ scores evaluated by parents and teachers of children who survived the tsunami changed with time after this severe natural disaster. However, the main hypothesis was rejected after careful analysis of the data.

Our results imply that after a major disaster, relying on a self-rating questionnaire as the screening tool to assess children's strengths and difficulties may result in an inflated number of children with emotional problems, conduct problems, hyperactivity/inattention, and peer relationship problems. Furthermore, not all children who survived this natural disaster exhibited reduction in their strengths in home activities. However, these children exhibited complication of their emotional problems, conduct problems, hyperactivity, and peer relationship problems at school and in home activities; moreover, their prosocial behaviors in school activities were undermined after the disaster.

These results indicate that prosocial behaviors such as caring, gentle, and honest toward their parents of children who survived the disaster (who had many problems) was not changed; however, that toward their friends and teachers at their school was changed after the disaster. It is necessary to discuss that differences between the prosocial behaviors scored by teachers and that scored by parents related with their individual problems such as traumatic experiences, single mother family, low academic achievements, disrupting class, and rebelliousness toward adults. Clinicians should take care to engage teachers after a disaster by consulting with them, as well as with parents, to care for child survivors of the disaster.

After any disaster, PTSD should be considered most carefully. The PTSD diagnostic criteria included in DSM-5 specify that patients experience significant difficulties in their daily lives because of their post-traumatic symptoms [14]–[23]. However, a previous study reported that the burden faced by parents and teachers when handling child survivors was not significantly relative with the children's post-traumatic symptoms and that the difficulties between children and parents and children and teachers are significantly different [2]. Our results and those of a previous study may be explained by the fact that parents take care of children at home, whereas teachers interact with children only at school, i.e., at different times of the day and with a different duration of interaction. Therefore, the children assessed here would not have been diagnosed with PTSD. Our findings are expected to improve the diagnosis of PTSD in pediatric populations, especially among children affected by natural disasters.

### Limitations

This study was a survey that used a self-rating questionnaire and was conducted in only one district in Japan; it is impossible to calculate the morbidity of PTSD in children after the 2011 Japanese earthquake and tsunami based on the results of this survey. Therefore, this study is inadequate as an epidemiological survey to establish a psychiatric diagnosis. Examinations by child psychiatrists using operational diagnostic criteria and structured interviews are necessary for accurate psychiatric diagnoses. In addition, the results of this study of children in Ishinomaki City do not reflect all the characteristics of the children who experienced the 2011 Japanese earthquake and tsunami.

Furthermore, this study used published normal SDQ data. The study of the normal data had been conducted before 2011 Japan earthquake and tsunami. However, these normal data cannot eliminate the effects of other disaster or children's individual traumatic experiences. Therefore, it is necessary to discuss the relationships between the normal SDQ data and traumatic experiences.

## Conclusions

This study showed that, the experience of the disaster affected those children with emotional and conduct problems at home and school, and with prosocial behaviors towards teachers and friends at school. However, no significant changes (in their prosocial attitude) had been seen at home, where they continued to keep their respect and caring feelings for parents. The difficulties experienced while handling these children were significantly greater for the parents than for the teachers. This indicates that clinicians should not only evaluate these children's daily activities at home but also try to objectively assess their daily activities at school in order to establish an accurate diagnosis.
